# Somatic loss of estrogen receptor beta and p53 synergize to induce breast tumorigenesis

**DOI:** 10.1186/s13058-017-0872-z

**Published:** 2017-07-03

**Authors:** Igor Bado, Fotis Nikolos, Gayani Rajapaksa, Wanfu Wu, Jessica Castaneda, Savitri Krishnamurthy, Paul Webb, Jan-Åke Gustafsson, Christoforos Thomas

**Affiliations:** 1 Department of Biology and Biochemistry, Center for Nuclear Receptors and Cell Signaling, University of Houston, 3517 Cullen Blvd, Houston, TX 77204 USA; 2 Department of Pathology, The University of Texas MD Anderson Cancer Center, 1515 Holcombe Blvd, Houston, TX 77030 USA; 3 Department of Genomic Medicine, Houston Methodist Research Institute, Houston Methodist, 6670 Bertner Avenue, Houston, TX 77030 USA

**Keywords:** Estrogen receptor beta, Breast cancer, p53, Genetically engineered mice, Breast carcinogenesis

## Abstract

**Background:**

Upregulation of estrogen receptor beta (ERβ) in breast cancer cells is associated with epithelial maintenance, decreased proliferation and invasion, and a reduction in the expression of the receptor has been observed in invasive breast tumors. However, proof of an association between loss of ERβ and breast carcinogenesis is still missing.

**Methods:**

To study the role of ERβ in breast oncogenesis, we generated mouse conditional mutants with specific inactivation of ERβ and p53 in the mammary gland epithelium. For epithelium-specific knockout of ERβ and p53, *ERβ* ^*F/F*^ and *p53* ^*F/F*^ mice were crossed to transgenic mice that express the Cre recombinase under the control of the human keratin 14 promoter.

**Results:**

Somatic loss of ERβ significantly accelerated formation of p53-deficient mammary tumors. Loss of the receptor also resulted in the development of less differentiated carcinomas with stronger spindle cell morphology and decreased expression of luminal epithelial markers.

**Conclusions:**

Our results show that synergism between ERβ and p53 inactivation functions to determine important aspects of breast oncogenesis and cancer progression.

**Electronic supplementary material:**

The online version of this article (doi:10.1186/s13058-017-0872-z) contains supplementary material, which is available to authorized users.

## Background

Estrogen signaling plays an important role in etiology and pathogenesis of breast cancer. In addition, estrogen receptors (ERs) have been associated with patient outcome in this particular disease. While ERα is an established molecular biomarker used for the selection of patients benefiting from endocrine therapy, the prognostic and predictive role of ERβ still remains controversial [1]. Despite the lack of verified association with patient course, a decline of ERβ expression in invasive tumors has been interpreted as a hallmark of tumor suppressor action in breast cancer [2–4]. More consistent evidence of the tumor-repressive activities of ERβ has been derived from in-vitro models of breast cancer. These include regulation of cellular response to DNA damage [5], induction of apoptosis [6], delayed cell cycle progression and inhibition of xenograft growth [7, 8], mesenchymal to epithelial transition, decreased cell migration/invasion and repression of oncogenic growth factor receptor and mutant p53 signaling [9–11].

Despite the effects of ERβ in cultured cells, whether the receptor elicits tumor repressive actions in a model system that reflects the etiology of human breast cancer is largely unknown. Analysis of ERβ knockout (KO) mice that were generated by employing different strategies to disrupt the ERβ gene showed a normal ductal growth but decreased side branching and partial alveolar development compared with the wild-type (WT) mice [12, 13]. Despite the incomplete differentiation of the mammary epithelium, conventional ERβKO mice were not prone to spontaneous breast tumor formation [12, 13]. To investigate the role of ERβ in breast cancer development and progression, we generated mice carrying conditional *ERβ* and/or *p53* alleles and a *Cre* transgene which is regulated by the human K14 promoter that is active in several epithelial tissues including the mammary gland epithelium [14–17]. We used mice with conditional *ERβ*-null alleles (*K14CreERβ* ^*F/F*^) to examine the impact of somatic loss of ERβ in breast oncogenesis that reflects the reduced expression of the receptor in human tumors and to avoid the effects of ovarian dysfunction with irregular cycle and hormone production that was observed in conventional ERβ knockouts [12, 13]. We selected the *p53* conditional-mutant mice because of the frequent inactivation of the tumor suppressor pathway in breast tumors [18]. In addition, because mammary tumors in *K14Crep53* ^*F/F*^ mice arise after a relatively long latency period, this model is suitable for investigating phenotypic consequences of additional alterations involved in tumor onset and progression [16]. The potential collaboration of ERβ loss of function and p53 inactivation in breast carcinogenesis is supported by in-vitro and in-vivo studies showing interactions between estrogen and p53 signaling in breast cancer. Loss of p53 has been suggested to synergize with estrogen to induce breast cancer [17, 19]. This synergism may reflect the molecular associations of p53 with ERs that occur in normal mammary and breast cancer cells. Indeed, ERα was found to bind to and repress the transcriptional activity and tumor suppressor function of p53 [20, 21]. On the other hand, an interaction of ERβ with mutant p53 has been shown to result in less invasive cellular phenotypes [11, 22].

Here, we present in-vivo evidence that loss of ERβ in mammary epithelial cells shortens the latency of p53-deficient tumors and results in tumors with displayed spindle cell morphology. Our study suggests the contribution of *ERβ* loss of function in mammary tumorigenesis and provides a valuable mouse model to delineate the functions of ERβ in breast cancer biology and therapy.

## Methods

### Mouse lines

The *p53* ^*F/****F***^ mouse strain was obtained from Dr Berns’ laboratory (Netherlands Cancer Institute) and maintained on a C57BL/6 J genetic background [16]. The *ERβ* ^*F/F*^ mouse strain described previously was also maintained on a C57BL/6 J background [23]. The *K14Cre* mice in a mixed background (STOCK Tg(KRT14-cre)1Amc/J, stock #004782; The Jackson Laboratory) were backcrossed to the C57BL/6 J background for a total of four generations, with the final two backcrosses followed by a genome scan that verified above 97% C57BL/6 J congenicity. The mice with the highest percentage of C57BL/6 J background were selected for the next generation of breeding. *K14Cre* mice which express Cre recombinase in several epithelial tissues including the mammary gland epithelium were crossed to *p53* ^*F/****F***^ and *ERβ* ^*F/F*^ animals to generate *K14Crep53* ^*F/****F***^ and *K14CreERβ* ^*F/F*^ mice in which *p53* and *ERβ* are deleted in the epithelium. To introduce the *ERβ* ^*F*^ allele into the *K14Crep53* ^*F/****F***^ model, we intercrossed *ERβ* ^*F/F*^ mice with *K14Crep53* ^*F/F*^ mice to produce *K14CreERβ* ^*F/F*^ *p53* ^*F/F*^ and *K14CreERβ* ^*F/+*^ *p53* ^*F/F*^ females. All mice were bred and maintained in the American Association for Accreditation of Laboratory Animal Care-approved Houston Methodist Research Institute Animal Care Facility in compliance with the approval from the institution animal protocol.

### Genotyping

To distinguish the *K14Cre* mice, ear and tail-tip DNA was analyzed by PCR with the primers oIMR1084 and oIMR1085 (The Jackson Laboratory) that yield a 100-bp product. All primer sequences used in genotyping are presented in Additional file 1: Table S1. Presence of the *ERβ* ^*F*^ allele was detected by PCR amplification of the *loxP* site in intron 3 that yields products of 160 and 300 bp for the wild-type and floxed alleles, respectively [23]. The *p53* ^*F*^ allele was detected as described previously [15]. Following amplification of the *loxP* site in intron 1, PCR products of 370 and 288 bp indicate the floxed and wild-type alleles, respectively. Fragments of 584 and 431 bp indicate the floxed and wild-type alleles after amplification of the *loxP* site in intron 10.

### RNA extraction and reverse transcription PCR

Frozen normal mammary gland and tumor tissues were disrupted and homogenized in Qiazol lysis reagent (Qiagen). Total mRNA was isolated using the RNeasy mini kit (Qiagen). RNA was reversed transcribed to cDNA using the iScript™ cDNA Synthesis Kit (Biorad). Reverse transcription (RT) PCR for the detection of the *ERβ* mutant allele was carried out either with primers that amplify a 173-bp product (exon 3) or with primers in exons 2 and 5 that amplify a wild-type transcript (430 bp) and a shorter transcript (259 bp) lacking exon 3 and analyzed in 2% agarose gel by electrophoresis. For the detection of loss of *p53* allele, mRNA was analyzed by RT-PCR with a set of primers in *p53* exons 10 and 11. The sequences of primers used for the RT-PCR experiments are presented in Additional file 1: Table S1.

### Histology and immunohistochemistry

Tissues were collected and fixed in 4% paraformaldehyde for 48 h. Tissues were embedded in Histowax, cut into 4-μm sections and stained with hematoxylin and eosin (H&E) for histopathological evaluation. For immunostaining, antigen retrieval was performed in citrate buffer (pH 6) for 10 min with the PT module and endogenous peroxidases were blocked by incubating the sections in 0.3% H_2_O_2_ at room temperature for 30 min. The sections were incubated with primary antibodies against ERβ (anti-ERβ 503 antibody that recognizes the C-terminus of the receptor), CK8 (TROMA-1C; DSHB), E-cadherin (Clone 35; BD Biosciences), ERα (Clone 60C; Millipore) and α-SMA (Clone 1A4; Sigma). Antibodies against Vimentin (D21H3) and the active form of B-catenin (D2U8Y) were from Cell Signaling. The antibodies against CK14 (LL002), p63 (ERP5701) and Ki-67 were from Abcam and N-cadherin was from Novus Biologicals. Sections were stained with biotin-conjugated secondary antibodies, followed by ABC reagent (Vactastatin ABC kit; Vector Laboratories) for the HRP-conjugated avidin–biotin complex. Peroxidase activity was visualized using 3,3′-diaminobenzidine (DAKO and Thermo Scientific) as a chromogen.

### Statistical analysis

The log-rank test was used to identify statistically significant differences in the tumor-free survival period. Statistical significance was obtained when *P* < 0.05.

## Results

### Conditional inactivation of ERβ in the mammary gland epithelium

Despite the generation of several mouse strains with conventional knockout of ERβ, no good model to assess the role of ERβ in breast tumorigenesis has so far been developed. Conventional deletion of exon 3 of ERβ was associated with deficient mammary gland development, but was not sufficient to induce breast cancer [12, 13, 23]. Thus, we sought to investigate the consequences of somatic loss of ERβ in a mouse mammary tumor model based on epithelium-specific inactivation of p53. We made use of the *ERβ* ^*F/F*^ mice that are homozygous for *ERβ* alleles in which the Cre/LoxP system (loxP sites in introns 2 and 3) was designed to target the exon 3 which encodes the first zinc finger of the ERβ DNA binding domain (DBD) [23]. For epithelial inactivation of the receptor, *ERβ* ^*F/F*^ mice were crossed to transgenic mice that express the Cre recombinase under the control of the human keratin 14 promoter (*K14Cre*). *K14Cre* mice express the Cre recombinase in several epithelial tissues including the skin and mammary gland epithelium. In the mammary gland, analysis of a reporter mouse line revealed K14Cre recombinase activity in both luminal epithelial and myoepithelial cells [15]. Activation of the Cre deleter in *ERβ* ^*F/F*^ mice results in the removal of exon 3 of ERβ which through a frameshift of the reading frame creates two in-frame stop codons and prevents translation of any transcript downstream of exon 3. A previous study reported the expression of an ERβ truncated protein despite the presence of the two stop codons in mice carrying conditional *ERβ* alleles and the CMV-Cre transgene [23].

**Fig. 1:**
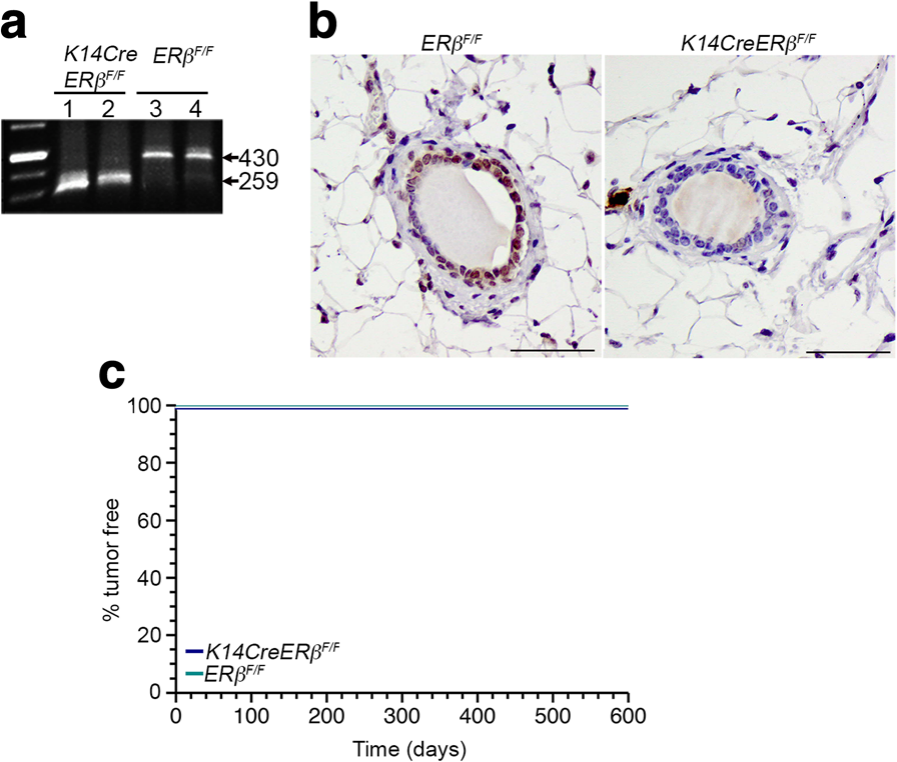
K14Cre-mediated conditional inactivation of ERβ alone does not predispose to mammary tumors. **a** RT-PCR analysis of total RNA from mammary glands of 8-week-old *K14CreERβ* ^*F/F*^ and *ERβ* ^*F/F*^ (control) mice with primers that amplify a sequence which spans exons 3 and 4 of ERβ cDNA. Mammary glands from control mice express a 430-bp ERβ mRNA whereas those from *K14CreERβ* ^*F/F*^ mice express a shorter transcript (259 bp) that lacks exon 3. Ablation of exon 3 was shown previously to induce splicing between exons 2 and 4 which through a frameshift in the open reading frame creates two in-frame stop codons. **b** Immunohistochemical analysis of mammary glands from *K14CreERβ* ^*F/F*^ and *ERβ* ^*F/F*^ mice for ERβ expression. ERβ staining is seen predominantly in nuclei of epithelial cells and in some myoepithelial cells. *Scale bars*, 50 μm. **c** Incidence of spontaneous mammary tumors in *K14CreERβ* ^*F/F*^ and *ERβ* ^*F/F*^ mice. Kaplan–Meier mammary tumor-free mouse survival curves for *K14CreERβ* ^*F/F*^ (*blue*; *n* = 14) and *ERβ* ^*F/F*^ (*green*; *n* = 4) females. *ER* estrogen receptor (Color figure online)

To determine whether ERβ was efficiently deleted in the epithelium of the mammary gland in *K14CreERβ* ^*F/F*^ mice, we analyzed mRNA from mammary glands of 8-week-old female *ERβ* ^*F/F*^ and *K14CreERβ* ^*F/F*^ mice by RT-PCR using primers that amplify a sequence which spans exons 3 and 4 of ERβ [23]. As shown in Fig 1a, the presence of the shorter *ERβ* transcript in the mammary glands of *K14CreERβ* ^*F/F*^ mice implies splicing between exons 2 and 4 that results in ablation of exon 3. Immunohistochemical analysis of the same tissues with an antibody that recognizes an epitope in the C-terminus of ERβ verified the lack of the receptor protein in luminal epithelial and myoepithelial cells of the mammary gland of the *K14CreERβ* ^*F/F*^ mice (Fig. 1b). To investigate whether K14Cre-mediated conditional inactivation of ERβ alone in the mammary epithelium predisposes to cancer, we monitored *K14CreERβ* ^*F/F*^ and *ERβ* ^*F/F*^ female mice for tumor formation during a 600-day period. As shown in Fig. 1c, none of the animals developed mammary tumors during this period.

### Loss of ERβ and p53 synergize in mouse mammary tumorigenesis

Given that estrogen was previously found to synergize with p53 inactivation on breast carcinogenesis [17, 19], we set out to develop a mouse breast tumor model based on epithelium-specific inactivation of ERβ and p53. We made use of mice carrying conditional p53 alleles (*p53* ^*F/F*^) in which loxP sites were inserted in introns 1 and 10 to avoid the development of lymphomas and sarcomas that are predominant in conventional p53 knockouts [24]. We crossed the *p53* ^*F/F*^ animals to *K14Cre* transgenic mice. In the resulting *K14Crep53* ^*F/F*^ animals, Cre recombinase excises exons 2–10 and through frameshift of the coding region prevents the synthesis of functional p53 protein [15].

**Fig. 2:**
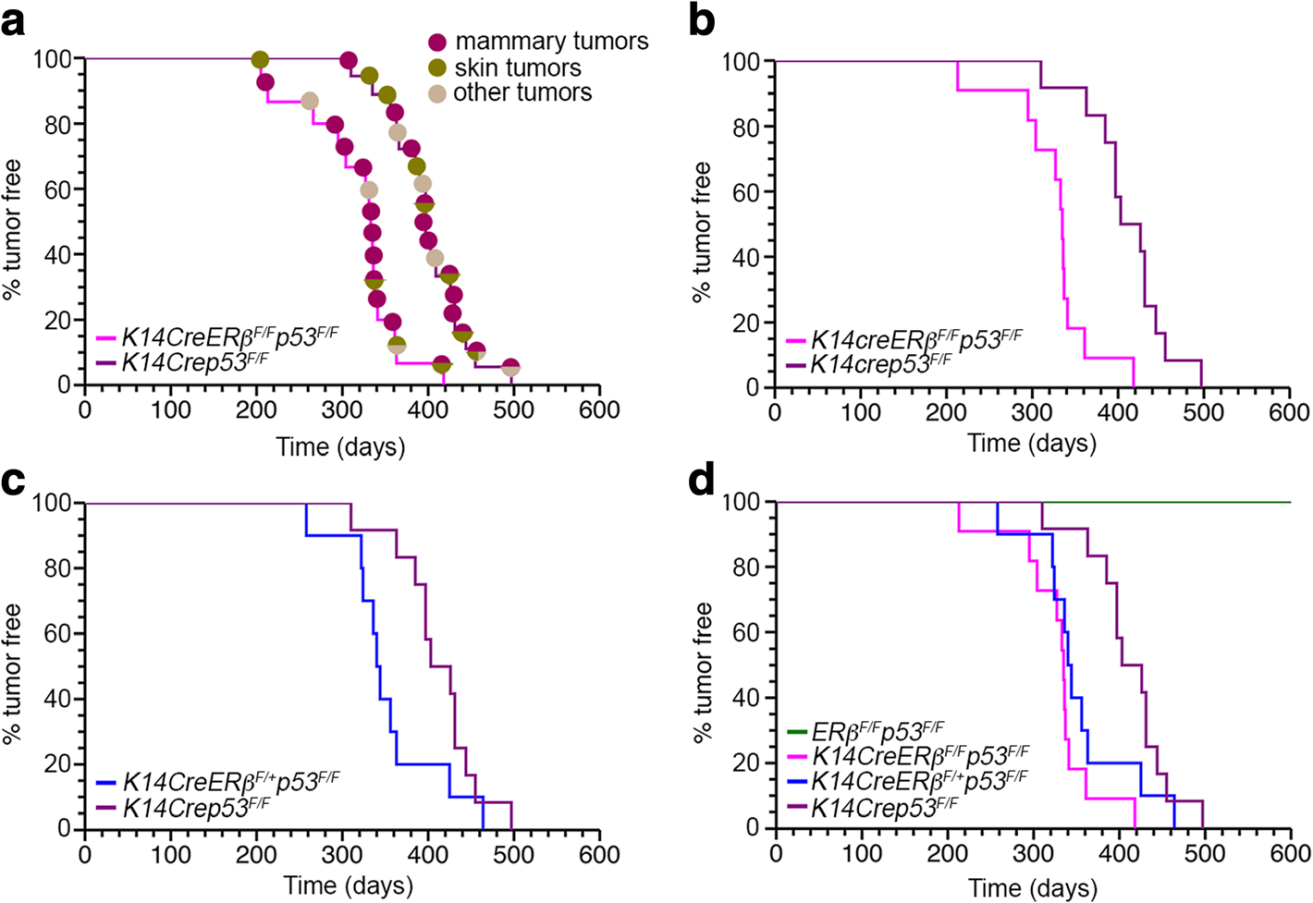
Cancer incidence and spectrum of tumors in *K14Cre* mice carrying conditional alleles of *ERβ* and *p53*. **a** Kaplan–Meier curves of tumor-free survival in *K14CreERβ* ^*F/F*^ *p53* ^*F/F*^ (*magenta*; *n* = 15) and *K14Crep53* ^*F/F*^ (*dark magenta*; *n* = 18) female mice. Median time of tumor-free survival (*T*_50_) for *K14Crep53* ^*F/F*^ females is 397 days and that for *K14CreERβ* ^*F/F*^ *p53* ^*F/F*^ females is 333 days (*P* < 0.0001). Tumor types (mammary, skin or other) indicated for each mouse. **b** Kaplan–Meier mammary tumor-free *K14CreERβ* ^*F/F*^ *53* ^*F/F*^ (*magenta*; *n* = 11; *T*_50_ = 335 days) and *K14Crep53* ^*F/F*^ (*dark magenta*; *n* = 12, *T*_50_ = 403 days) females (*P* = 0.00066). **c** Kaplan–Meier mammary tumor-free curve of *K14Crep53* ^*F/F*^ female mice (*dark magenta*) vs *K14CreERβ* ^*F/+*^ *p53* ^*F/F*^ female mice (*blue*; *n* = 10; *T*_50_ = 337 days; *P* = 0.06). **d** Mammary tumor-free mouse survival curves of *K14Crep53* ^*F/F*^ (*dark magenta*), *K14CreERβ* ^*F/F*^ *p53* ^*F/F*^ (*magenta*), *K14CreERβ* ^*F/+*^ *p53* ^*F/F*^ (*blue*) and *ERβ* ^*F/F*^ *p53* ^*F/F*^ (*green*; *n* = 16) female mice. Mice were killed when mammary tumors reached a diameter of approximately 1 cm for mammary tumors or approximately 0.8 cm for skin tumors. *ER* estrogen receptor (Color figure online)

Previous studies showed that *K14Crep53* ^*F/F*^ mice develop mammary carcinomas and carcinosarcomas with a median latency period of 330 days [14, 16]. The same mice also develop a smaller number of skin tumors and occasionally or rarely tumors in other Cre-expressing tissues such as the salivary gland and jaws [14, 16]. Treatment of *K14Crep53* ^*F/F*^ mice with exogenous estrogen in another study shortened the time of onset of mammary tumors [17]. In our study, we monitored *K14Crep53* ^*F/F*^ mice for the development of spontaneous neoplasms for a 20-month period and observed a similar spectrum of tumors with those that were observed previously in the absence of exogenous estrogen (Fig. 2a). Consistent with previous studies, mammary tumors were developed in 66% of *K14Crep53* ^*F/F*^ mice [16]. The same mice developed skin tumors with a significantly lower frequency (38%) while tumors in salivary glands and jaws were rare occurrences (Fig. 2a). Our *K14Crep53* ^*F/F*^ female mice on a C57BL/6 J genetic background developed mammary and skin tumors with a median latency of 397 days.

**Fig. 3:**
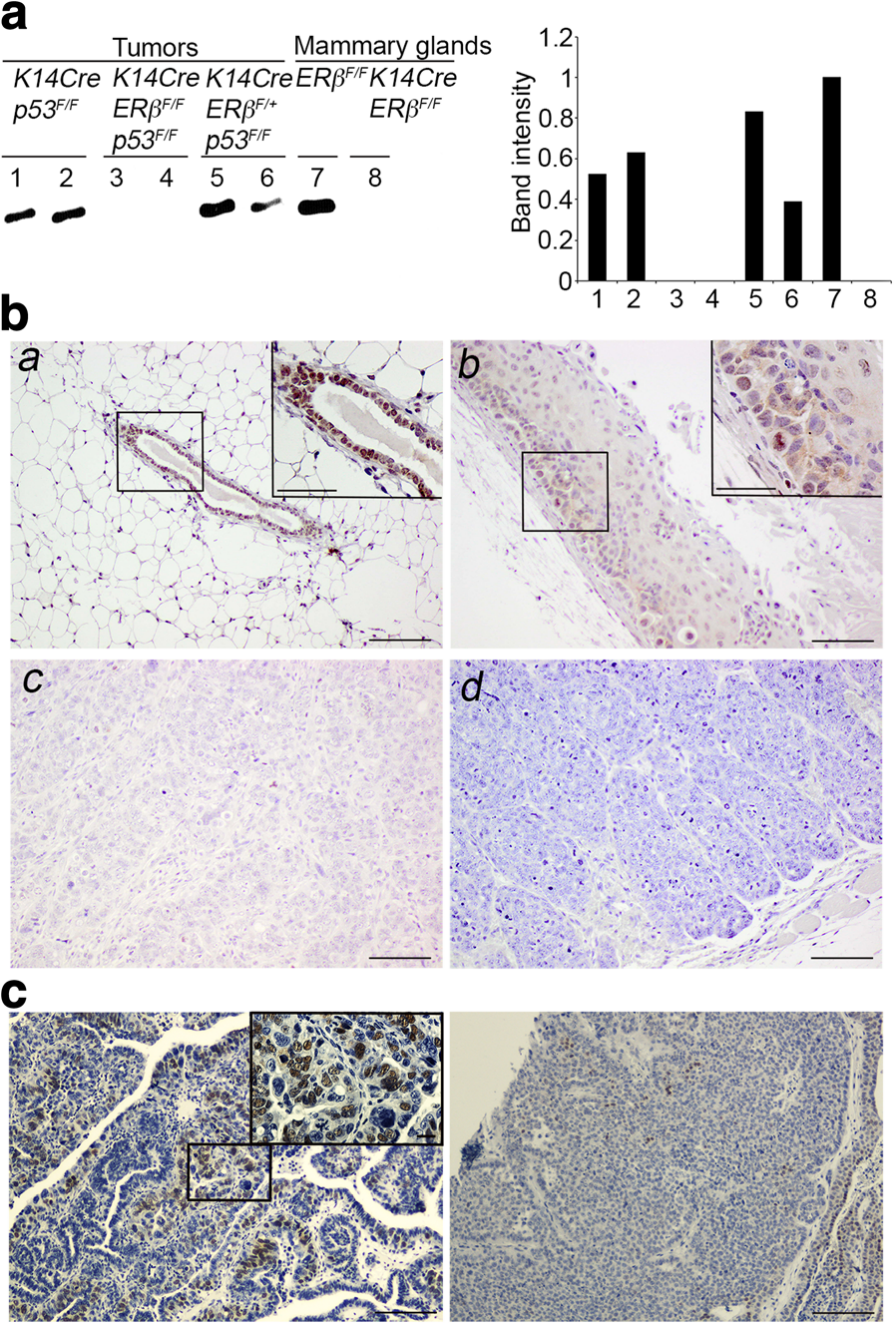
Expression of ERβ in normal mammary gland and p53-deficient mammary tumors. **a** RT-PCR analysis of total RNA from mammary glands of 8-week-old *K14CreERβ* ^*F/F*^ and *ERβ* ^*F/F*^ (control) female mice as well as mammary tumors from *K14Crep53* ^*F/F*^ and *K14CreERβ* ^*F/F*^ *p53* ^*F/F*^ female mice with primers that amplify the exon 3 of ERβ cDNA. Of note, a decline of ERβ expression during tumor development is demonstrated by the decreased levels of the ERβ transcript in p53-deficient mammary tumors of *K14Crep53* ^*F/F*^ mice compared with the normal mammary glands of the control mice (lane 7 vs lanes 1 and 2). Graph represents quantification of the band intensities of ERβ exon 3 cDNA in RT-PCR analysis (normalized to that of the normal mammary gland in lane 7). **b** Sections containing normal mammary gland of 8-week-old *ERβ* ^*F/F*^ female mouse (*a*) and mammary tumors from *K14Crep53* ^*F/F*^ (*b*, *c*) and *K14CreERβ* ^*F/F*^ *p53* ^*F/F*^ (*d*) female mice were stained for ERβ. Nuclei of the epithelial cells of the normal duct present strong staining (*a*). *Inset image* displays enlargement of the denoted duct. *Scale bars*, 100 μm (inset 50 μm). Microphotographs of tumor sections *b* and *c* are from the same ERβ-positive tumor from *K14Crep53* ^*F/F*^ mice (developed in the absence of p53). Compared with the mammary gland (*a*), there is weaker staining in the nuclei of the tumor epithelial cells (*b*) consistent with the difference in ERβ mRNA levels in **a**. In addition, within the same tumor, there is reduced expression of ERβ in poorly differentiated epithelial cells (*c*) compared with the tumor component with more intact cell polarity (*b*). Lack of ERβ expression is observed in mammary tumors from *K14CreERβ* ^*F/F*^ *p53* ^*F/F*^ mice (*d*). **c** Breast tumor cells with ERα-positive nuclei in *K14Crep53* ^*F/F*^ (*left*) and *K14CreERβ* ^*F/F*^ *p53* ^*F/F*^ (*right*) mice. *Inset image* shows enlargement of the denoted tumor area. *Scale bars*, 100 μm. *ER* estrogen receptor

To investigate whether loss of ERβ synergizes with p53 inactivation to promote early onset of breast tumors, we introduced the *ERβ* ^*F*^ allele into the *K14Crep53* ^*F/F*^ model to generate *K14CreERβ* ^*F/F*^ *p53* ^*F/F*^ and *K14CreERβ* ^*F/+*^ *p53* ^*F/F*^ female mice. Compared with *K14Crep53* ^*F/F*^ animals, *K14CreERβ* ^*F/F*^ *p53* ^*F/F*^ female mice developed mostly mammary and a small number of skin tumors with a significantly reduced median latency of 333 days (Fig. 2a; *P* < 0.0001). In these mice, mammary tumors arose between 213 and 418 days, indicating a relatively uniform tumor development (Fig. 2b). In addition to a shortened latency, the proportion of female mice that developed mammary tumors was higher in the *K14CreERβ* ^*F/F*^ *p53* ^*F/F*^ group (74%) compared with the *K14Crep53* ^*F/F*^ cohort (66%), suggesting that loss of ERβ is specifically associated with mammary tumorigenesis. It is also noted that tumors from *K14CreERβ* ^*F/F*^ *p53* ^*F/F*^ mice grew slightly faster compared with those in *K14Crep53* ^*F/F*^ animals (37 vs 42 days until maximum allowable size); however, the difference was not statistically significant. Compared with the *K14CreERβ* ^*F/F*^ *p53* ^*F/F*^ mice, the median mammary tumor-free survival period of *K14CreERβ* ^*F/+*^ *p53* ^*F/F*^ mice was noticeably shorter than that of *K14Crep53* ^*F/F*^ animals; however, the difference did not reach statistical significance, indicating that loss of both ERβ alleles might be necessary to enhance tumorigenesis in these mice (Fig 2c, d; *P* = 0.06). RT-PCR analysis revealed comparable levels of the ERβ transcript in some tumors of *K14CreERβ* ^*F/+*^ *p53* ^*F/F*^ mice and in tumors from *K14Crep53* ^*F/F*^ animals, which may account for the lack of significant difference in tumor latency between the two cohorts (Fig. 3A). In addition to tumor onset, differences were observed in the number of mammary tumors that were formed in different groups of mice. While all *K14Crep53* ^*F/F*^ mice developed single mammary tumors, a number (3/21; 15%) of *K14CreERβ* ^*F/F*^ *p53* ^*F/F*^ and *K14CreERβ* ^*F/+*^ *p53* ^*F/F*^ mice developed multiple breast cancers.

### Reduced expression of ERβ in p53-deficient mammary tumors

A gradual decline, but not a complete loss, of ERβ expression was observed previously during the transition from normal breast to ductal hyperplasia and from carcinoma in situ to invasive cancers [2–4, 25]. *K14Crep53* ^*F/F*^ mice were shown previously to develop tumors with ERα-negative cells in the absence of exogenous estrogen and often ERα-positive tumors after treatment with estrogen [16, 17]. ERα-positive tumors also developed under the conditional inactivation of p53 by the WAP Cre linking the expression of ERα in mammary tumors to the time of p53 inactivation during mammary gland development [26]. We investigated whether mammary tumors from *K14Crep53* ^*F/F*^ mice retain the expression of ERβ. As shown in Fig. 3A, RT-PCR analysis indicates decreased levels of an ERβ transcript in mammary tumors from *K14Crep53* ^*F/F*^ mice compared with the normal mammary gland of the *p53* ^*F/F*^ mice (Fig. 3A). Immunohistochemical analysis revealed that a subset (5/9; 55%) of the mammary tumors from the *K14Crep53* ^*F/F*^ mice retained ERβ expression. However, the levels of the receptor in positive tumors were lower compared with those in the epithelial cells that line the ductal system of the normal breast (Fig. 3B *a*, *b*). In addition, the expression of ERβ was not uniform within the same tumor. Reduced expression of the receptor was detected in poorly differentiated tumor epithelial cells with disrupted cell polarity compared with the tumor component with more intact cell organization (Fig. 3B *b*, *c*). RT-PCR analysis also indicated different expression of ERβ across tumors from heterozygous mice for the conditional *ERβ* allele (Fig 3A). Compared with *K14Crep53* ^*F/F*^ mice, not all mammary tumors from *K14CreERβ* ^*F/+*^ *p53* ^*F/F*^ mice displayed decreased levels of the ERβ transcript, which may indicate stochastic recombination of the single conditional ERβ allele, apparently due to low activity of the K14Cre in the mammary gland. The heterogeneous expression of the receptor across *K14CreERβ* ^*F/+*^ *p53* ^*F/F*^-derived tumors may account for the variation in tumor latency that was observed in mice of this group (Fig. 2c, d). In addition, similar to previous studies that analyzed *K14Crep53* ^*F/F*^ mice, a few mammary tumors displayed ERα-positive nuclei and there was no significant difference in ERα expression in tumors between *K14Crep53* ^*F/F*^ and *K14CreERβ* ^*F/F*^ *p53* ^*F/F*^ mice (Fig. 3C) [15].

### Histological characteristics of mammary tumors in *K14Crep53*^*F/F*^ and *K14CreERβ*^*F/F*^*p53*^*F/F*^ female mice

**Fig. 4:**
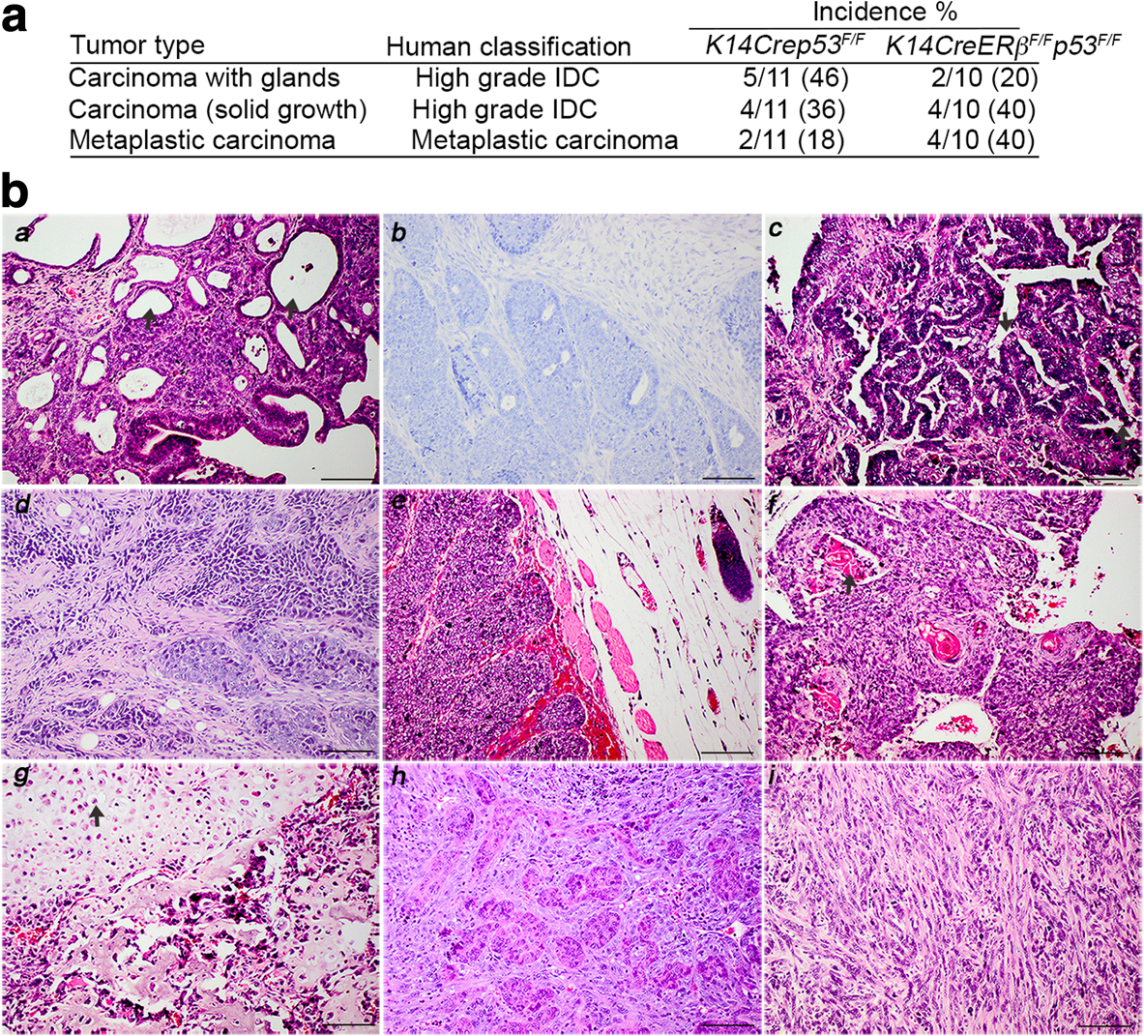
Spectrum, incidence and histopathology of mammary tumors from *K14Crep53* ^*F/F*^ and *K14CreERβ* ^*F/F*^ *p53* ^*F/F*^ female mice. **a** Incidence of different breast cancer types from *K14Crep53* ^*F/F*^ and *K14CreERβ* ^*F/F*^ *p53* ^*F/F*^ female mice and their human classification. **b** Histopathology of mammary tumors derived from *K14Crep53* ^*F/F*^ and *K14CreERβ* ^*F/F*^ *p53* ^*F/F*^ female mice. Histological sections of representative tumors were stained with H&E. (*a*, *b*) Carcinoma with glandular differentiation (*arrows* indicate gland formation). (*c*) Papillary carcinoma with well-defined borders and small finger-like projections (indicated by *arrows*). (*d*, *e*) Poorly differentiated carcinoma defined by expansive growth. Tumors in *a–e* resemble human high-grade IDC. (*f*) Poorly differentiated metaplastic carcinoma showing squamous differentiation centrally in nests (*arrow* shows keratin deposits). (*g*) Poorly differentiated matrix producing metaplastic carcinoma that contains a myxoid matrix with large cells with chondroid appearance (indicated by *arrows*). (*h*, *i*) Poorly differentiated carcinoma with spindle cell metaplasia consisting of either epithelial and mesenchymal components (biphasic carcinoma) (*h*) or largely of spindle cells arranged in crossing bundles (*i*). Tumors in *h* and *i* resemble carcinosarcomas in humans. *Scale bars*, 100 μm. *ER* estrogen receptor, *IDC* invasive ductal carcinoma

*K14Crep53* ^*F/F*^ female mice were shown previously to develop two types of mammary tumors. These include pure epithelial tumors with either glandular differentiation or without glands and an expansive growth pattern that resemble human invasive ductal carcinoma (IDC) and biphasic tumors with both epithelial and mesenchymal elements that resemble human carcinosarcomas [14, 16]. Similar to these findings, *K14Crep53* ^*F/F*^ mice in our study developed epithelial tumors with or without glands and tumors with spindle cell metaplasia (Fig. 4). Compared with the *K14Crep53* ^*F/F*^ mice, *K14CreERβ* ^*F/F*^ *p53* ^*F/F*^ female mice developed less epithelial tumors with glandular differentiation (20% vs 46%) and a higher proportion of poorly differentiated tumors with metaplastic histology and spindle cell morphology (40% vs 18%) (Fig. 4A, B). Of note, a mammary tumor from *K14CreERβ* ^*F/+*^ *p53* ^*F****/F***^ mice displayed features of a primary osteosarcoma/matrix producing metaplastic carcinoma with a myxoid matrix containing large cells with chondroid appearance defined by hyperchromatic nuclei and vacuolated cytoplasm (Fig. 4B). In addition to differences in histology, *K14CreERβ* ^*F/F*^ *p53* ^*F****/F***^-derived mammary tumors showed a higher proliferation index compared with tumors from *K14Crep53* ^*F/F*^ mice as indicated by the expression of the proliferation marker Ki-67, which may account for their slightly higher growth rate (Additional file 2: Figure S1).

### Loss of *ERβ* results in tumors with mesenchymal and EMT phenotypes

**Fig. 5:**
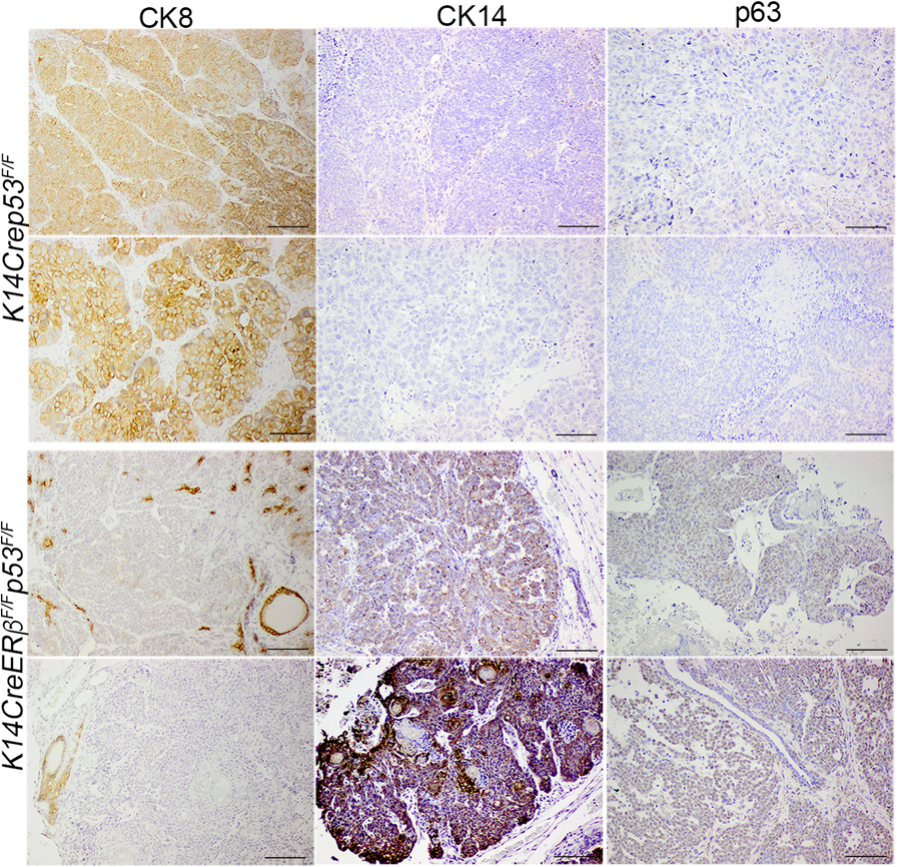
Inactivation of ERβ induces basal breast tumor phenotypes. Representative tumor sections from *K14Crep53* ^*F/F*^ and *K14CreERβ* ^*F/F*^ *p53* ^*F/F*^ female mice were stained with antibodies against cytokeratin 8 (*CK8*), cytokeratin 14 (*CK14*) and p63. Distinct expression patterns of luminal and basal markers were observed between mammary tumors from the two groups of mice. *Scale bars*, 100 μm. *ER* estrogen receptor

**Fig. 6:**
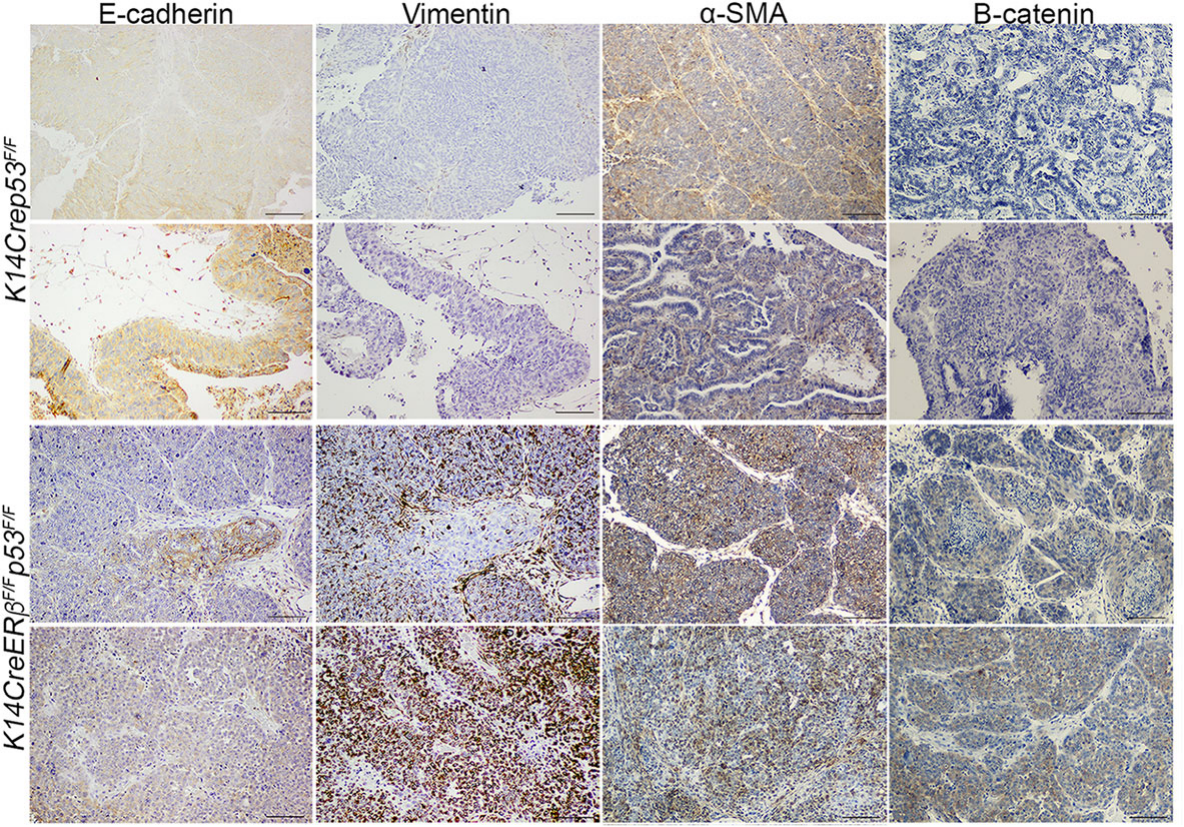
Loss of ERβ is associated with occurrence of EMT in mammary tumors. Microphotographs of serial tumor sections analyzed by immunohistochemistry for the expression of E-cadherin, vimentin, α-SMA and the cytoplasmic active form of B-catenin. Areas of tumors arising in the absence of ERβ (*K14CreERβ* ^*F/F*^ *p53* ^*F/F*^ mice) display decreased levels or loss of E-cadherin and increased expression of vimentin, α-SMA and B-catenin. *Scale bars*, 100 μm. *ER* estrogen receptor, *α-SMA* alpha-smooth muscle actin

*K14Crep53* ^*F/F*^ mice develop tumors that are similar to human sporadic basal-like breast cancers [16]. Upregulation of ERβ in basal-like breast cancer cell lines has been shown to induce an epithelial transformation which results in decreased cell migration and invasion [9–11]. Compared with *K14Crep53* ^*F/F*^ mice, a higher fraction of tumors with spindle cell morphology arose in the absence of ERβ in *K14CreERβ* ^*F/F*^ *p53* ^*F/****F***^ mice, suggesting that the expression of the receptor may be required for epithelial maintenance during tumor development (Fig. 4). To corroborate this association, we analyzed mammary tumors for the expression of luminal and basal cell markers by immunohistochemistry. As shown in Fig. 5 (top left and middle panels), a higher number of mammary tumors in *K14Crep53* ^*F/F*^ mice displayed a large CK8-positive (10/10; 100%) and CK14-negative (6/10; 60%) cell type. In contrast, a higher proportion of mammary tumors in *K14CreERβ* ^*F/F*^ *p53* ^*F/****F***^ mice showed sporadic expression of CK8 and strong expression of CK14 (8/9; 89%) (Fig. 5, bottom left and middle panels). In addition to CK14, the other basal cell marker p63 was expressed in the majority of tumors from *K14CreERβ* ^*F/F*^ *p53* ^*F/****F***^ mice (7/8; 87%) (Fig. 5, bottom right panel). Given the presence of epithelial and mesenchymal cell types in tumors of *K14CreERβ* ^*F/F*^ *p53* ^*F/****F***^ mice, we sought to determine whether EMT occurs in these tumors. It is known that during EMT expression of E-cadherin decreases and expression of vimentin, alpha-smooth muscle actin (α-SMA) and N-cadherin increases [27, 28]. In addition, B-catenin is localized in the cell membrane of epithelial cells and its active form is translocated to the cytoplasm and nucleus of cells that undergo EMT [28]. As shown in Fig. 6 and Additional file 3: Figure S2, an increased number of mammary tumors that were developed in the absence of ERβ expressed vimentin (7/10; 70%), α-SMA (7/9; 77%), the cytoplasmic active form of B-catenin (7/7; 100%) and N-cadherin (8/9; 88%) or displayed a loss of E-cadherin (6/10; 60%) compared with the *K14Crep53* ^*F/F*^ mice (vimentin (3/10; 30%), α-SMA (3/9; 33%), B-catenin (0/7; 0%) and N-cadherin (4/9; 44%)). The tumors with strong expression of vimentin from *K14CreERβ* ^*F/F*^ *p53* ^*F/****F***^ mice developed with significantly reduced latency (330 days) and were less differentiated compared with the tumors with low vimentin expression from *K14Crep53* ^*F/F*^ mice (416 days, *P* < 0.001), suggesting that occurrence of EMT in the absence of ERβ may be associated with earlier onset of nonwell-differentiated breast cancers. These results strengthen the notion that ERβ is essential for epithelial maintenance during the development of the mammary gland and in breast cancer.

## Discussion

Despite the controversy that exists over the clinical importance of ERβ in breast cancer, evidence from in-vitro and clinical studies points toward a role as a tumor suppressor in this malignancy. Loss of ERβ expression has been observed during the development and progression of breast cancer and increased levels of the receptor in normal gland with decreased risk of developing the disease [2–4, 29–31]. Moreover, upregulation of ERβ in breast cancer cells is associated with enhanced apoptosis and decreased invasion [5, 9]. However, a proof of the link between the loss of ERβ function and breast cancer is lacking. Previous studies showed that conventional inactivation of ERβ alone does not predispose to mammary tumors [12, 13]. Thus, to investigate the effect of ERβ loss in breast carcinogenesis, we created a mouse model for breast cancer in which somatic mutations of p53 and ERβ are targeted in K14-expressing mammary epithelial cells. p53 loss of function is a frequent event in breast cancer [18]. On the other hand, Keratin 14 in puberty and in adulthood marks basal/myoepithelial cells which as long-lived bipotent stem cells expand to both luminal and basal cell populations and play important role in ductal morphogenesis and ductal homeostasis [32]. Thus, inactivation of tumor suppressor genes in these actively dividing cells using the K14Cre-loxP technology not only makes them likely targets for malignant transformation, but also determines the type of cancer that could develop later. p53 has been suggested to control the quality and quantity of stem cells to enable proper development and prevent cancer. Increase in the long-termed regenerative mammary stem cells has been observed in p53^–/–^ mice [33, 34]. In addition, treatment with estrogen has been associated with increased mammary stem cell activity of [35]. The effects of estrogen and p53 on mammary stem cells may reflect the synergism between the hormone and p53 loss that was shown to induce early onset of mammary tumors [17]. However, the mechanism underlying the interaction between estrogen and p53 signaling in breast carcinogenesis is still not well defined. Estrogen binds to both ERs but the differential expression of ER subtypes in different cell types and developmental stages determines the effects of hormone in the development of the target tissue. We observed expression of ERβ in luminal epithelial and myoepithelial cells in the mammary ducts of wild-type mice at the end of puberty. We expected that if ERβ functions as a tumor suppressor, its inactivation in mammary stem cells by the K14Cre recombinase would influence aspects of breast oncogenesis. Such impact of ERβ could be detected in a breast cancer model based on epithelium-specific inactivation of p53 in which mammary tumors developed with a relatively long median latency period [14, 16, 17].

As in the conventional ERβ knockout mice, K14Cre-mediated conditional inactivation of ERβ in the mammary gland alone does not lead to tumor development [12, 13]. In contrast, analysis of the *K14CreERβ* ^*F/F*^ *p53* ^*F/****F***^ mouse model provides in-vivo evidence of an effective synergism between ERβ and p53 tumor suppressor function in genesis and progression of breast cancer. The tumor repressive activity of ERβ in this model is manifested by the accelerated tumor development in *K14CreERβ* ^*F/F*^ *p53* ^*F/****F***^ mice as compared with the *K14Crep53* ^*F/F*^ mice. The contribution of loss of ERβ function in tumorigenesis in this animal model may be associated with the antiproliferative activity of the receptor [36, 37]. An increase in rounds of precancerous cell division that is induced by ERβ deletion may synergize with an increased mutation rate that is driven by the absence of p53. Such functional collaboration between ERβ loss with p53 inactivation can increase the probability of acquiring mutations that result in tumor development with shorter latency.

In addition to early-onset tumors, loss of ERβ leads to a more undifferentiated tumor phenotype with increased expression of basal epithelial and mesenchymal markers. Furthermore, the same decreased dosage of ERβ gene increases the proportion of tumors with features of EMT, suggesting that loss of the receptor is associated with more aggressive neoplasms and cancer cells that have higher potential to invade and metastasize to distant sites. This is in agreement with the epithelial and less invasive phenotype that has been consistently observed after upregulation of the receptor in breast cancer cells [9–11, 38, 39]. The preponderance of tumors with both myoepithelial and luminal cell types in the absence of ERβ suggests the involvement of multipotent progenitor cells in tumor development. K14-expressing basal/myoepithelial cells produce clones that consist of basal and/or luminal cell lineages. The cellular composition of mixed clones is skewed over time in a physiological adulthood toward the luminal-rich clones [32]. Deletion of the essential of the epithelial differentiation ERβ in precancerous mammary stem cells may lead to a shift toward more basal-rich clones which could result in more basal-like breast cancer.

We have generated a mouse model that demonstrates for the first time effects of ERβ inactivation in breast tumorigenesis. Our results show that loss of ERβ function acts in collaboration with p53 inactivation to affect several aspects of breast carcinogenesis including tumor initiation and progression. Concomitant loss of ERβ and p53 induces early onset of mammary tumors with more basal-like characteristics. This mouse model may assist in the identification of the molecular mechanisms employed by ERβ to elicit its tumor repressive actions in the breast and lead to development of treatment strategies to prevent breast cancer.

## Conclusions

In this study, we present in-vivo evidence that loss of ERβ function acts in collaboration with p53 inactivation to induce early onset of mammary tumors with spindle cell morphology and more basal-like characteristics. Our study demonstrates for the first time effects of ERβ inactivation in breast tumorigenesis and provides a valuable mouse model for delineating the tumor-repressive actions of ERβ in the breast and testing chemoprevention strategies.

## Additional files

Additional file 1: Table S1. Presenting oligonucleotides used in genotyping and RT-PCR. (PDF 209 kb)

Additional file 2: Figure S1. Showing proliferation characteristics of mammary tumors from *K14Crep53* ^*F/F*^ and *K14CreERβ* ^*F/F*^ *53* ^*F/F*^ female mice. **A** Microphotographs of tumor sections after staining with an antibody against the proliferation marker Ki-67. *Scale bars*, 100 μm. **B** Percentage of Ki-67-positive cells in a series of mammary tumors from *K14Crep53* ^*F/F*^ and *K14CreERβ* ^*F/F*^ *p53* ^*F/F*^ female mice. (PDF 2502 kb)

Additional file 3: Figure S2. Showing expression of N-cadherin in mammary tumors from *K14Crep53* ^*F/F*^ and *K14CreERβ* ^*F/F*^ *53* ^*F/F*^ female mice. Representative tumor sections from *K14Crep53* ^*F/F*^ and *K14CreERβ* ^*F/F*^ *p53* ^*F/F*^ female mice after staining with an antibody against N-cadherin. *Scale bars*, 100 μm. (PDF 2261 kb)

## Abbreviations

CK: Cytokeratin
DBD: DNA binding domain
EMT: Epithelial to mesenchymal transition
ERα: Estrogen receptor alpha
ERβ: Estrogen receptor beta
IDC: Invasive ductal carcinoma
KO: Knockout
RT-PCR: Reverse transcription PCR
WT: Wild-type
α-SMA: Alpha-smooth muscle actin
