# Supplementary material for: Somatic loss of estrogen receptor beta and p53 synergize to induce breast tumorigenesis
Source: Breast Cancer Res. 2017 Jul 3;19:79. doi: 10.1186/s13058-017-0872-z (PMC5494907; doi:10.1186/s13058-017-0872-z)
Supplement: Supplementary file 1 — Presenting oligonucleotides used in genotyping and RT-PCR. (PDF 209 kb) [file 13058_2017_872_MOESM1_ESM.pdf]

Table S1

Oligonucleotides used in genotyping (in 5' to 3' direction)

ERβ	
ISP	AAAGGTATGCTTATGTCCGGTGGG
BIASP	GTGGATGCCTATGATCACTGTGGA
p53	
p53-int1-FW	CACAAAAACAGGTTAAACCCAG
p53-int1-RV	AGCACATAGGAGGCAGAGAC
p53-int10-FW	AAGGGGTATGAGGGACAAGG
p53-int10-RV	GAAGACAGAAAAGGGGAGGG
K14Cre	
oIMR1084	GCGGTCTGGCAGTAAAAACTATC
oIMR1085	GTGAAACAGCATTGCTGTCACTT

Oligonucleotides used in RT-PCR

ERβ exon 2 FW	GCCAATCATCGCTTCTCTAT
ERβ exon 5 RV	GGCACTTCTCTGTCTTCGTA
ERβ exon 3 FW	GCCAGCCCTGTTACTAGTCC
ERβ exon 3 RV	TCACAGGACCAGACACCGTA
ERβ exon 3 FW	GGTTGTGCCAGCCCTGTTA
ERβ exon 3 RV	CAGGACCAGACACCGTAATGA
P53 FW	ACTCCAGCTACCTGAAGACCA
P53 RV	ACCCTATGAGGGCCCAAGAT
