# Supplementary material for: Somatic loss of estrogen receptor beta and p53 synergize to induce breast tumorigenesis
Source: Breast Cancer Res. 2017 Jul 3;19:79. doi: 10.1186/s13058-017-0872-z (PMC5494907; doi:10.1186/s13058-017-0872-z)
Supplement: Supplementary file 2 — Showing proliferation characteristics of mammary tumors from K14Crep53 F/F and K14CreERβ F/F 53 F/F female mice. A Microphotographs of tumor sections after staining with an antibody against the proliferation marker Ki-67. Scale bars, 100 μm. B Percentage of Ki-67-positive cells in a series of mammary tumors from K14Crep53 F/F and K14CreERβ F/F p53 F/F female mice. (PDF 2502 kb) [file 13058_2017_872_MOESM2_ESM.pdf]

Figure S1

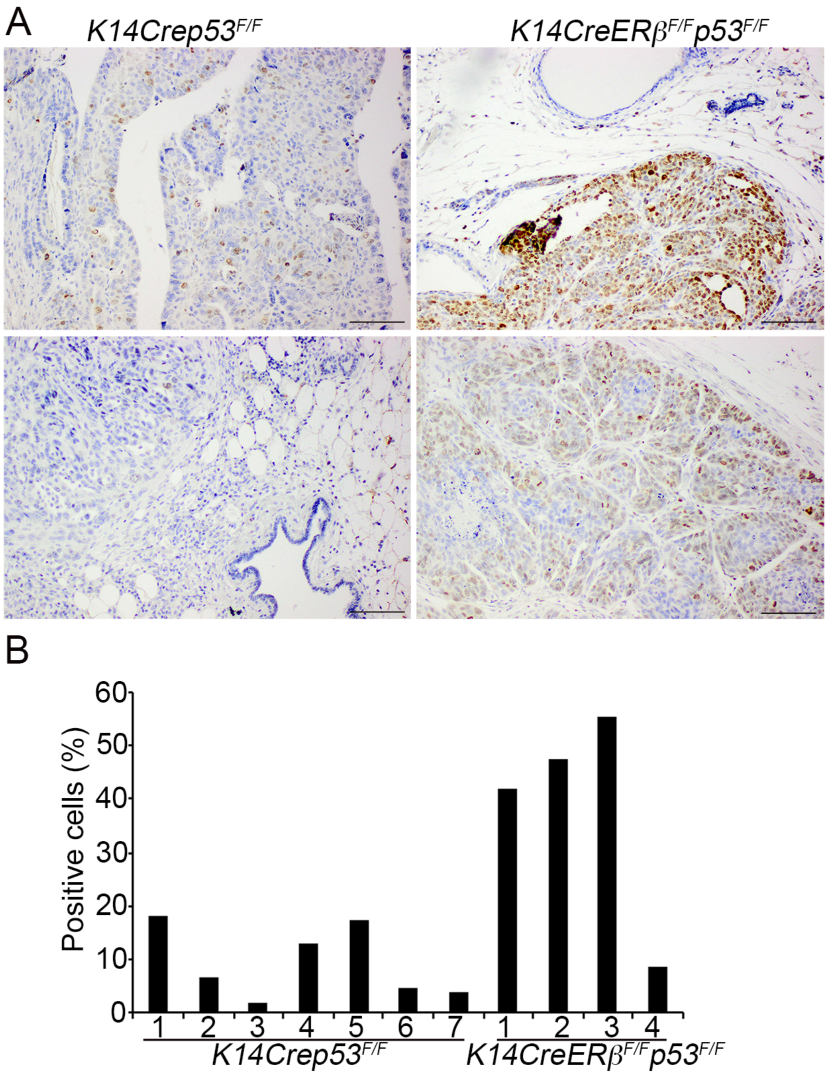


**Supplementary Figure 1. Proliferation characteristics of mammary tumors from *K14Crep53*$^{F/F}$ and *K14CreERβ*$^{F/F}$*53*$^{F/F}$ female mice.** (A) Microphotographs of tumor sections after staining with an antibody against the proliferation marker Ki-67. Scale bars 100 μm. (B) The graph shows the percentage of Ki-67-positive cells in a series of mammary tumors from *K14Crep53*$^{F/F}$ and *K14CreERβ*$^{F/F}$*p53*$^{F/F}$ female mice.
