# Supplementary material for: Somatic loss of estrogen receptor beta and p53 synergize to induce breast tumorigenesis
Source: Breast Cancer Res. 2017 Jul 3;19:79. doi: 10.1186/s13058-017-0872-z (PMC5494907; doi:10.1186/s13058-017-0872-z)
Supplement: Supplementary file 3 — Showing expression of N-cadherin in mammary tumors from K14Crep53 F/F and K14CreERβ F/F 53 F/F female mice. Representative tumor sections from K14Crep53 F/F and K14CreERβ F/F p53 F/F female mice after staining with an antibody against N-cadherin. Scale bars, 100 μm. (PDF 2261 kb) [file 13058_2017_872_MOESM3_ESM.pdf]

Figure S2

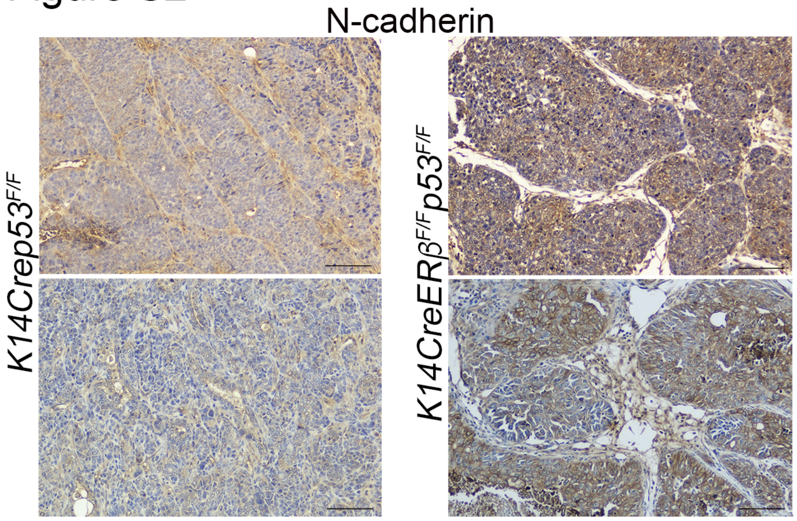

Supplementary Figure 2. Expression of N-cadherin in mammary tumors from *K14Crep53$^{F/F}$* and *K14CreERβ$^{F/F}$53$^{F/F}$* female mice. Representative tumor sections from *K14Crep53$^{F/F}$* and *K14CreERβ$^{F/F}$p53$^{F/F}$* female mice were stained with an antibody against N-cadherin. Scale bars 100 µm.
